# Reviews and Book Notices

**Published:** 1877-11

**Authors:** 


### Reviews and Book Notices.

A Series of American Clinical Lectures. Edited by *E. C. Seguin, M. D.* New York: *G. P. Putnam's Sons.* 1876.

Vol. II. No. V. (whole No. 17), Diagnosis of those Diseases of the Eye, which can be Seen without the Ophthalmoscope. By Henry D. Noyes, M. D., Professor of Ophthalmology and Otology, etc.

No. VI. (18), The Modern Methods of Examining the Upper Air Passages. By George M. Lefferts, M. D., Clinical Professor of Laryngoscopy, etc.

No. VII. (19), On Tracheotomy and Laryngotomy. By H. B. Sands, M. D., Professor of Anatomy, etc.

No. VIII. (20), The Hypertrophied Prostate. By Robert F. Weir, M. D., Lecturer on Genito-Urinary Diseases, etc.

No. IX. (21), Points in the Surgery of Childhood. By J. H. Pooley, M. D., Professor of Surgery, etc.

No. X. (22), Spinal Irritation; Its Pathology and Treatment. By William A. Hammond, M. D., Professor of Diseases of the Mind and Nervous System, etc.

No. XI. (23), On the Treatment of Eczema. By R. W. Taylor, M. D., Physician to Charity Hospital, New York.

Vol. III. No. I. (25), Transfusion of Blood and its Practical Applications. By Thomas G. Morton, M.D., one of the Surgeons of the Pennsylvania Hospital, etc.

Clinical Lectures, delivered by men of recognized ability, are always attractive. They possess a charm of their own. Their very freedom from the abstract and abstruse reasoning of the didactic teacher, their application of principle to practice, of the methods of diagnosis to the case to be diagnosticated, of the remedy to the condition to be remedied, are attractive merits. The conversational tone of the writing, while it might detract from the dignity of a more studied effort, is here not without its value. Nothing is more detestable than a slovenly-prepared clinical lecture. He who spreads before his auditors (or readers) a clumsily compounded abstract of the text-books he has chanced to consult upon the subject in hand, as well as he who laboriously enlarges upon a topic which has been worn threadbare by every tyro in medicine, is equally an offender with the ignorant or careless author.

All of the merits, and few, if any, of the faults described, distinguish this valuable series of essays; and the editor has fairly earned the honor of congratulation, not only on account of the practical results here displayed, but also on account of the brilliancy of the scheme itself. The lectures are, as a rule, characterized by originality, conciseness, accuracy and practical suggestions. Some of them are quite equal in point of classical and literary excellence to the productions of that prince of clinical exposition—the eloquent teacher of the Hotel-Dieu de Paris.

Dr. Noyes' Lecture, (No. V.), treats exclusively of the question of *diagnosis* of such ocular and orbital diseases as can be recognized without the aid of the opththalmoscope, and by the employment merely of oblique illumination through the medium of a convex lens of two-inch focal distance. This subject will thus commend itself especially to the general practitioner, whose attention to diseases of the eye too frequently terminates, when it is thought to be necessary to employ the ophthalmoscope. All lesions possible of recognition without this instrument, from imperfect accommodation to those of traumatic origin, are here concisely described with a view to their recognition.

Dr. Leffert's Lecture, (No. VI.), will prove chiefly useful to those who are unfamiliar with the use of the laryngoscope, since it points out clearly the chief causes of failure. The usual obstacles are enumerated as follows: a short frenum linguæ, an unmanageable tongue, irritability of the fauces, hypertrophy of the tonsils, elongation of the uvula, and an unfavorable position of the epiglottis. A wood-cut produces very distinctly the normal relation of all the parts represented in the laryngeal picture upon the mirror. The essentials for examination are stated to be, two mirrors, one laryngoscopic, one rhinoscopic, a forehead reflector, (preferably provided with Kramer's band) good illumination, if possible by artificial reflected light, and one Türk's spatula.

Dr. Sands (Lecture, No. VII.), in the enumeration of the morbid conditions of the air passages which may require the artificial opening of the wind-pipe, mentions the various forms of laryngitis, as well as foreign bodies in the air passages, laryngeal or tracheal tumors, injuries of the larynx or trachea, stenosis of the larynx after wounds or ulcers, and spasm or paralysis of the laryngeal muscles. We find no allusion here to membranoid occlusions, which the author, without doubt, would class under the division of specific laryngitis, as in the greater number of instances these membranous formations result from syphilitic disease. The morbid conditions external to the air passages, which may call for the operation, are thus given: impaction of foreign bodies in the pharynx, or œsophagus; tumors overlying the superior aperture of the larynx; cervical tumors or abscesses pressing on the trachea; thoracic aneurisms pressing on the pneumogastric or recurrent laryngeal nerves, or other tumors having the same result; and lastly, operations in the mouth or pharynx, involving copious hemorrhage.

Dr. Weir's Lecture (No. VIII.), is a thoroughly careful and judicious exposition of the subject of prostatic hypertrophy, for the relief of the symptoms attending which, it is almost needless to say, diurnal catheterization is recommended. He advances beyond the teachings of the text-books, in pointing out the fact that it is muscular and glandular hyperplasia which induces the enlargement (myoadenoma), not, as has been taught, undue development of fibrous tissue. He gives a list of catheters found useful by himself in relieving the distension resulting from prostatic enlargement, but omits to mention two, either of which may prove effective when all others fail. One is the instrument made of cable wire, such as the dentists use. It is far more flexible than the vertebrated catheter, and should always be preferred to the latter in view of the danger which might result from the disconnection of its various links.

The other is the simple soft rubber instrument, open at one extremity and closed at the other, with foramina at the side. It can be insinuated where English and French instruments will fail to pass.

Dr. Pooley (Lecture No. IX.), has confined himself to a discussion of the method of operating in phimosis and hare-lip. His subject is treated in a judicious and practical manner, the lecturer evidently having enjoyed a fair experience in treating the deformities under discussion, but as the operations for relief of the latter are not, in any sense, exclusively performed in childhood, we are at a loss to perceive the fitness of the title of the lecture.

Dr. Pooley, however, while he traverses a well worn path, arouses a new interest at almost every step, by his ingenuity and candor. We have heretofore, in these pages, briefly noted his employment of black sutures after excision of the prepuce, in order to commend the proceedure as one of those little measures whose adoption often contributes to the elegance of the result.

Dr. Hammond (Lecture X.), understands by spinal irritation, that condition in which there is anæmia of the posterior columns of the spinal cord, or, as he terms it, "posterior spinal anæmia." An enumeration of the symptoms of a disease so exactly located would possess great interest in connection with this subject, but after brief reference to some of the former—the list including such widely divergent phenomena as hiccup, insanity, gastric acidity, asthma, visual disturbances, and incontinence of urine—the author disappoints us by stating that all have not been mentioned, since their detail would "take up more time than our lecture affords." The result is, that the reader of this essay does not gain a clear conception of the disease which the author is attempting to discuss, and, if the dim suspicion is awakened that there is room for doubt whether all these symptoms result from the pathological condition named, certainly only Dr. Hammond is to be blamed for it.

The treatment recommended is hygienic, onic, and the employment of remedies which increase the amount of blood in the spinal column, strychnia, phosphorus, opium, picrotoxine.

We should, however, not advise the too enthusiastic student of these clinical lectures, to employ the remedies named in "salivation," "somnambulism," "pyrosis," and "pain in the testicles," until he has become thoroughly familiar with all the symptoms of "posterior spinal anæmia."

Dr. Taylor's Lecture on the Treatment of Eczema (No. XI.), is one of the most delightful clinical essays of the series. He describes well the injurious results of applying tarry and caustic preparations in the local treatment of the disease in its acute phase, and praises the bran baths, and the application of alkaline lotions, with hot or very warm water, which are, as a rule, so grateful to the patient and so often efficacious in a remedial point of view.

The value of Dr. Taylor's injunction against the use of the iodide of potassium in such cases—a remedy to which the general practitioner is too often ready to resort—is well illustrated by a case which was made the subject of a clinical essay by Dr. Duhring, in the Hospital of the University of Philadelphia, and published in the *Philadelphia Medical and Surgical Reporter*, of August 4, 1877—since Dr. Taylor's lecture was put in print. In it a bullous eruption, induced by the iodide of potassium, had occurred extensively as a result of the improper treatment of a small patch of infiltrated eczema.

Lack of space forbids further reference to the treatment recommended in chronic forms of the disease, and especially to the local and internal treatment of eczema infantile, the therapeutic rules laid down being so clear, concise, and practical, that they cannot fail to be appreciated by the busy practitioner who would otherwise have to consult the larger treatises.

We believe that this lecture will prove of greater value to the average reader than any other of the series. And this, not merely because of the skill with which the author has handled the subject, but by reason of the fact that where one medical man, in general practice, is summoned to open the larynx, to operate for hair-lip, or to perform transfusion, twenty are asked what to do for a baby with "scalled head."

Dr. Martin's Lecture (No. III., No. I.), contains the following list of disorders for which transfusion has been considered available: hæmorrhage, idiopathic anæmia, chlorosis, leukæmia and exhaustion, pulmonary consumption, cholera; uræmic, phorphorus and carbonic oxide intoxication (of each of the two first named, one case has occurred and terminated favorably), carcinoma, pyæmia, septicæmia and puerperal fever, variola, scarlet fever and diphtheria, tetanus and epilepsy, scurvy, asphyxia neonatorum, hydrophobia, insanity, and sunstroke. Cuts are shown representing the apparatus of Howe, Aveling, and the author. The latter uses defibrinated blood, and his method is not peculiar to himself. He has occasionally injected into the radial artery toward the hand, after ligating above, but as a rule he considers the transfusion into a vein the better mode.

We conclude with a single extract: "I charge you to let no person die in an emergency, as from post-partum hæmorrhage, merely because you have not all the specified implements. A pocket case, a small syringe, and fresh blood, are all you will find necessary; and when great haste is required, I should not hesitate to inject the blood without defibrination. Only be reasonably caraful of clot and air, and especially avoid throwing in the blood rapidly. It can hardly be thrown in too slowly." J. N. H.

Some General Ideas Concerning Medical Reform. By *David Hunt, M. D.* A. Williams & Co., Boston. 1876.

This is a little book that deserves more of a notice than we have space for in this journal. In this day of rapid advancement in every department of thought, such books are valuable to tell us where we are, and whither we are drifting. It is plain, however, that the author, like so many others at the present day, has completely mistaken science for medicine. America might well be proud to have great anatomists, physiologists and pathologists. But anatomists, physiologists and pathologists are not physicians, however much they may contribute to improve the art. We would like to see the preliminary course of study so modified as to embrace the widest possible scientific education. We think we could lay out a course suited to the physician from ——— we were about to say from the *ovum* to the maturest manhood. We are quite sure, however, that the medical course proper, extending as it does over three years only, should be in the highest degree practical. This was probably the idea in establishing what is known as the "Summer School." By the most extensive clinical instruction it was purposed to teach young men *how to take care of sick people.* Every attempt to make medicine a science is an attempt to inaugurate or perpetuate a sort of medical monasticism. J. I. T.

THE CURE OF HERNIA—By what the Author terms New Methods—By *George Heaton, M. D.* Boston: H. O. Houghton & Co. 1877.

This book contains 196 pages neatly bound in cloth. The contents are arranged and edited by J. Henry Davenport, M. D., of Harvard.

The author devotes twenty-six pages to a description of what hernia is, and the different kinds of hernia; and very concisely and clearly describes the various forms of rupture, giving statistical reports from various sources showing the frequency of hernia in the human family.

The operations of Gerdy-Wutzer and Wood are not referred to, and the plan of plugging the hernial opening condemned, as causing too severe inflammatory action and the subsequent throwing out of the plug as a foreign body.

Then comes the author's method of cure, which is termed the "Liquid Method," or *the method of tendinous irritation.*

The operation is performed with a syringe somewhat resembling the ordinary subcutaneous syringe, which is loaded with the modicum of irritating fluid. The patient is placed in the recumbent position, and the contents of the hernia returned within the abdomen. The hernial sac also should be returned if possible.

Armed with the instrument charged and prepared, the operator introduces its beak into the inguinal canal. Supposing the case in question to be an inguinal hernia, care is taken not to injure the spermatic cord.

About ten minims of the liquid—which is made of the fluid extract of quercus alba, and of the solid alcoholic extract of the same material—is injected into the canal. Morphia is sometimes added to the solution to relieve pain.

A cut and description of the instruments used are shown in the book. After the operation a bandage or truss is worn, to retain the intestines or contents of the canal within the abdomen until a cure is completed.

The radical cure of the rupture is accomplished, as claimed by the author, by producing a low grade of inflammation in the fibrous or fibroid tissue causing lymph to be thrown out into the canal, which being thus subcutaneously produced, has a strong plastic or organizing tendency. Also that its production is largely interstitial, thickening the fascia and tendons, and thus closing the hernial or inguinal canal.

The theory is a plausible one, and the operation a comparatively simple one. It is a somewhat new method of trying to accomplish a result long since arrived at, but too seldom attained.

The author speaks confidently of the result, and the cures by this plan of treatment in his own hands, and we confess that his statements have evidence of candor as well as facts.

Get the book; it is well worth reading. And the operations for the cure of rupture are well worth trying. H. V.

Aiken as a Health Resort. By *Dr. W. C. Geddings, M. D.*

Dr. Geddings has been for several years a resident of Aiken and a careful student of the climate of this section of the Southern States. In this pamphlet of about 30 pages he has collected a large number of valuable facts, bearing upon the relation of climate to disease, and especially the climate of the sand hills of South California to diseases of the respiratory organs. His conclusions are that benefit may be reasonably expected in that climate in most chronic diseases of an asthenic type. He enumerates

1st. Bronchitis. 2d. Consumption, (except in last stages, and in acute tuberculosis, laryngeal phthisis). 3d. Malarial Disease. 4th. Dyspepsia. 5th. Anæmia. 6th. Diseases of Females. 7th. Diseases resulting from over work, confinement, etc. 8th. Convalescence from Pneumonia and Pleuritis. 9th. Convalescence from Typhoid Fevers, and other Exhausting Diseases. 10th. Syphilis. 11th. Children convalescing from Scarlatina, Measles, Whooping-cough, etc.

Aiken is contra-indicated in the following diseases.

1st. Laryngeal Consumption. 2d. Laryngitis. 3d. Bronchitis when attended with very tight cough, sparse secretion. 4th. Bright's Disease. 5th. Eye Diseases. 6th. Diseases of the Nervous System.

The reasons of these conclusions are indicated, but cannot be given for the want of space.

THE TONIC TREATMENT OF SYPHILIS. By *E. L. Keyes*, A. M., M. D., adjunct professor of surgery, etc., New York. *D. Appleton & Co.*, 1877, pp. 83.

The author of this interesting little monograph, published an article on the subject of blood counting with the hématimètre, in the *American Journal of the Medical Sciences* for January, 1876, which speedily attracted attention to his studies and secured for them a favorable recognition. The present treatise seems to have been largely the result of the investigations reported in the paper named above, the attempt being here made to establish upon a satisfactory basis a rational method of treating syphilis. We have, heretofore, in the digest department of this JOURNAL given a brief abstract of the conclusions reached by the author after repeated and careful experiments in counting the blood corpuscles of syphilitic patients—not only those who were untreated but also those who were brought under the influence of various medicaments. A fair illustration of the method of treatment preferably adopted by Dr. Keyes, as a result of experiments, may be found in his use of the centigramme granules of the protiodide of mercury, prepared by Messrs. Garnier & Lamoureux, of Paris.

For three days one granule is taken immediately after each meal, and on the fourth day one granule is added to the mid-day dose—this addition being continued for three days also. On the fourth day again, another granule is added (the patient, for example, taking two in the morning, one at noon, and two at night), and this course also is continued for three days. Such gradual addition is progressively conducted until there is positive evidence of intestinal irritation, as for example, colicky pain, diarrhœa, or symptoms of incipient salivation, when the patient's "*full dose*" has been ascertained. This is to be distinguished from his "*tonic dose*," which latter is held to be one-half of the former amount, and one which should be unceasingly administered, day after day and month after month, waiting for new symptoms. Upon the supervention of the latter, recurrence should be had to the "full dose," for their prompt relief.

Such is a brief outline of the scheme of what Dr. Keyes calls the "tonic treatment of syphilis." But the title has already given rise to a misapprehension on the part of the reviewers of the monograph, which has elicited a rejoinder from the author. In it[1] he takes occasion to utterly disclaim any responsibility for the argument that because mercury in small doses is tonic and when thus administered is capable of curing syphilis, therefore the drug is curative by virtue of its tonic action. On the contrary, he claims that mercury in short courses induces the disappearance of syphilitic symptoms, but produces debilitating effects if continued in full specific doses. Long continued in small doses, it controls the disease and is physiologically tonic. Consequently the specific action of mercury may be so managed as to constitute the remedy a tonic medicament.

This much for the distinctive features of this little book, which are certainly full of interest, suggestive and of great practical moment. It is true that the general system of procedure recommended, is by no means novel or untried, having been largely employed by successful practitioners before the author's experiments were instituted. He is, however, fully

1. Louisville Medical News, No. 10, April 28th, 1877, p. 199.

entitled to the credit of presenting it anew in a lucid and forcible manner, to the notice of the general profession, and, in particular, for his successful refutation of the somewhat hastily reached conclusions of Wilbouchewitch, Grassi and Ricord.

Apart from these distinctive peculiarities of the essay, a chapter is added upon the local treatment of especial lesions of syphilis, which are considered in a manner quite satisfactory, when the small space allotted to the subject is considered, neither the title nor scope of the volume, however, really demanding any such addition.

We are pleased to note that the claim of the hot springs ot Arkansas, with reference to the eradication of syphilis, is here justly characterized as "utterly unfounded in fact." Other denunciation might have been employed for the purpose of warning syphilitic patients of the shameless impositions of the charlatans who swarm in the vicinity of that resort. We must admit to some surprise, in reading, on page 61, a concession as to even the "adjuvant" value of guaiac and sarsaparilla in the treatment of the disease; and cannot but deprecate the use of such language as the following in connection with any scientific subject: "No means in the physician's hands place him so near the Deity as the iodide of potassium." Such words suggest, in a most uncomfortable manner, the extravagance of statement which usually accompanies the *suggestio falsi* or the *suppresio veri.*

The typographical appearance of the work is unexceptionable; and we believe that, as a whole, it will not fail to add to the established reputation of the adjunct professor of surgery in Bellevue Hospital Medical College. J. N. H.

